# Melatonin: Multi-Target Mechanism Against Diminished Ovarian Reserve Based on Network Pharmacology

**DOI:** 10.3389/fendo.2021.630504

**Published:** 2021-04-19

**Authors:** Liuqing Yang, Hongbin Xu, Yun Chen, Chenyun Miao, Ying Zhao, Yu Xing, Qin Zhang

**Affiliations:** ^1^ Department of Traditional Chinese Medical Gynecology, Hangzhou Hospital of Traditional Chinese Medicine Affiliated to Zhejiang Chinese Medical University, Hangzhou, China; ^2^ Second Clinical Medical College, Guangzhou University of Chinese Medicine, Guangzhou, China; ^3^ Dongfang Hospital, Beijing University of Chinese Medicine, Beijing, China

**Keywords:** diminished ovarian reserve (DOR), ovarian reserve (OR), melatonin, potential therapeutic targets, signaling pathways, biological processes, network pharmacology

## Abstract

**Background:**

Diminished ovarian reserve (DOR) significantly increases the risk of female infertility and contributes to reproductive technology failure. Recently, the role of melatonin in improving ovarian reserve (OR) has attracted widespread attention. However, details on the pharmacological targets and mechanisms of melatonin-improved OR remain unclear.

**Objective:**

A systems pharmacology strategy was proposed to elucidate the potential therapeutic mechanism of melatonin on DOR at the molecular, pathway, and network levels.

**Methods:**

The systems pharmacological approach consisted of target identification, data integration, network construction, bioinformatics analysis, and molecular docking.

**Results:**

From the molecular perspective, 26 potential therapeutic targets were identified. They participate in biological processes related to DOR development, such as reproductive structure development, epithelial cell proliferation, extrinsic apoptotic signaling pathway, PI3K signaling, among others. Eight hub targets (MAPK1, AKT1, EGFR, HRAS, SRC, ESR1, AR, and ALB) were identified. From the pathway level, 17 significant pathways, including the PI3K-Akt signaling pathway and the estrogen signaling pathway, were identified. In addition, the 17 signaling pathways interacted with the 26 potential therapeutic targets to form 4 functional modules. From the network point of view, by regulating five target subnetworks (aging, cell growth and death, development and regeneration, endocrine and immune systems), melatonin could exhibit anti-aging, anti-apoptosis, endocrine, and immune system regulation effects. The molecular docking results showed that melatonin bound well to all hub targets.

**Conclusion:**

This study systematically and intuitively illustrated the possible pharmacological mechanisms of OR improvement by melatonin through anti-aging, anti-apoptosis, endocrine, and immune system regulation effects.

## Introduction

Infertility affects a significant proportion of humanity and is regarded as a global public health issue by the World Health Organization (1, 2). Diminished ovarian reserve (DOR), defined as a reduction in both oocyte quality and quantity, is one of the most common causes of female infertility and poor ovarian response to controlled ovarian stimulation with a rapidly increasing occurrence rate (3, 4). In addition, women with DOR have exceedingly high rates of recurrent pregnancy loss and no euploid embryos (5–7). Devine et al. reported that the prevalence of DOR increased from 19 to 26% in the past few years, representing a major challenge in reproductive medicine (8, 9). Despite its prevalence, its pathology remains unclear. Aging is the most common cause of DOR. Other influential factors for DOR include genetic predisposition, autoimmune diseases, chemotherapy, and psychological stress (10–13).

The decline of ovarian reserve (OR) is a continuous, gradual process starting from the oocyte death of embryos at 20 weeks of gestation until menopause (14). The premature depletion of OR eventually results in premature ovarian failure, a more severe condition, which might lead to a loss of reproductive capacity, seriously affecting women's quality of life (15, 16). Thus, early and active interventions should be implemented in women with DOR before it is too late. However, DOR treatment remains a significant challenge in reproductive medicine, although various treatment strategies are currently being used (9). For example, DHEA, as an adjuvant therapy in *in vitro* fertilization (IVF), might increase the number of retrieved oocytes (17); however, the true benefit is under active debate as DHEA has some side effects, including acne, sleep problems, and headaches (18).

Melatonin (5-methoxy-N-acetyl tryptamine), a pineal gland hormone, plays a significant role in regulating the circadian sleep-wake cycle, reproductive physiology, and immune functions (19). As a dietary supplement, it has gained widespread popularity globally. Lerner and colleagues' discovery of melatonin in 1958 presented a new research avenue in reproductive physiology (20, 21). Since Wurtman et al. reported that preovulatory follicles contain substantial amounts of melatonin, which may affect ovarian steroidogenesis, many studies have focused on the role of melatonin in OR (21). Morioka et al. conducted the first clinical trial to evaluate melatonin as a drug for improving oocyte quality in women who could not become pregnant because of poor-quality oocytes (22). The results showed that melatonin treatment increased oocyte quality. Interestingly, the melatonin-treated group's intrafollicular melatonin concentration was four times higher than that of the control group, consistent with Morioka's study. Similarly, several subsequent studies have confirmed that melatonin supplementation can ameliorate intrafollicular oxidative balance, improve the quantity and quality of oocytes, and improve IVF outcomes in women with DOR and infertility (23–27). Some experts have suggested that melatonin levels in the follicular fluid may serve as a biomarker for predicting OR (28, 29). In addition, animal experiments have also confirmed that melatonin can protect the quality of oocytes and improve OR through multiple mechanisms (30–35).

**Figure 1:**
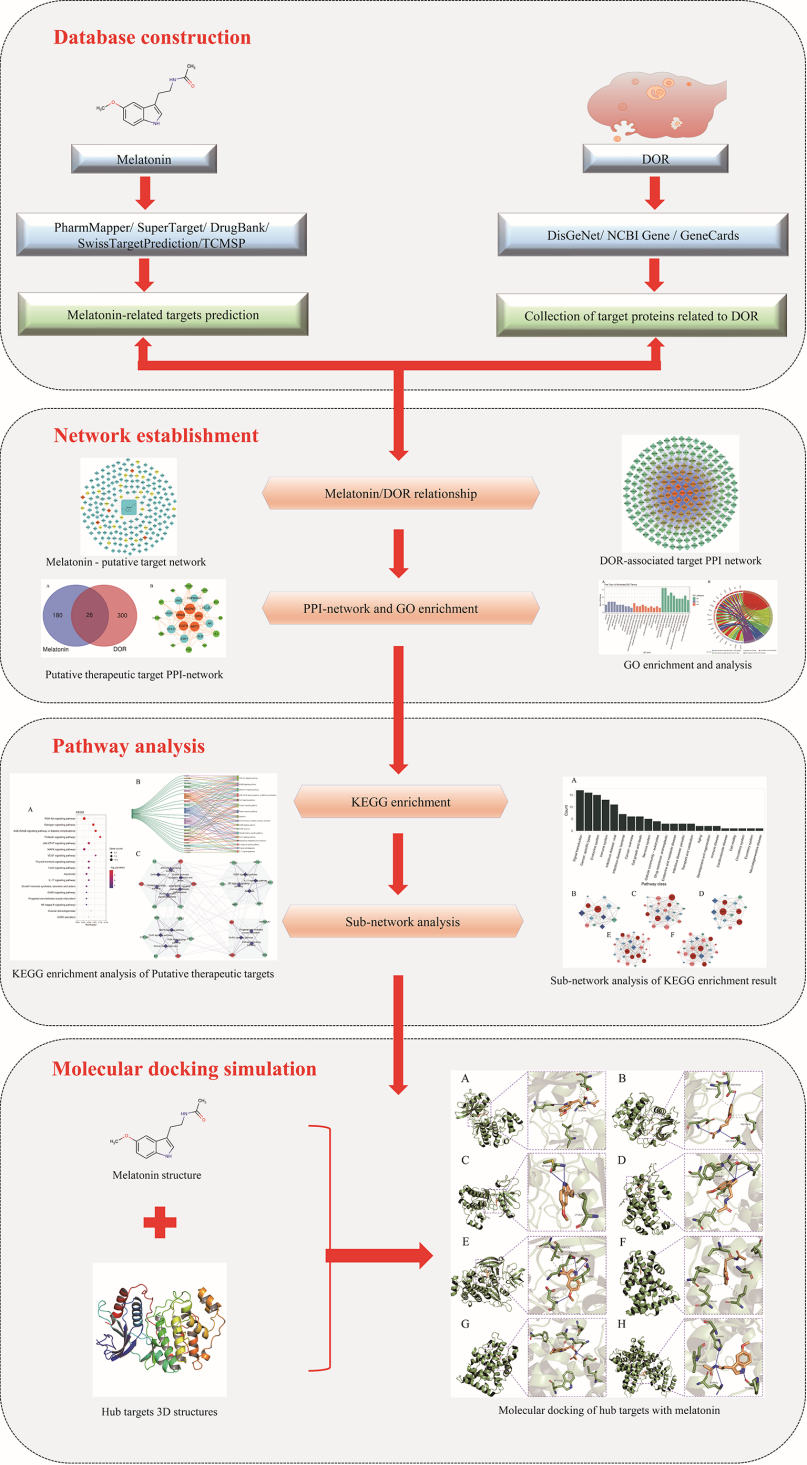
Research workflow diagram.

Although anti-DOR activities exerted by melatonin have been reported, in-depth mechanistic preclinical studies are currently limited. Moreover, details of biomarkers and the biological pathways through which melatonin exerts its effects in improving OR are yet to be completely elucidated. In a previous study, a network pharmacology-based approach was successfully used to uncover the target proteins and potential therapeutic mechanisms of drugs (36–38). Accordingly, this study was performed to reveal the predictive targets and therapeutic mechanisms underlying melatonin action against DOR using a systematic network pharmacology-based approach. **Figure 1** illustrates the workflow of the study.

## Materials and Methods

### Identification of Putative Melatonin Targets

The PubChem database (https://pubchem.ncbi.nlm.nih.gov/) was used to obtain simplified molecular-input line-entry specification (SMILES) information and the 3D structure of melatonin (39). Melatonin's 3D structure was uploaded to the PharmMapper Server (http://www.lilab-ecust.cn/pharmmapper/), and the SMILES for melatonin was uploaded to the SwissTargetPrediction database (http://www.swisstargetprediction.ch/) to predict the potential melatonin targets (40, 41). DrugBank (https://go.drugbank.com/), SuperTarget (http://insilico.charite.de/supertarget/index.php), and TCMSP (https://tcmspw.com/tcmsp.php) databases were used to identify known melatonin targets (42, 43). All retrieved target names were corrected to official symbols using the UniProt database(https://www.uniprot.org/).

### Selection of DOR-Associated Targets

DisGeNET (https://www.disgenet.org/), GeneCards (https://www.genecards.org/), and NCBI Gene databases (https://www.ncbi.nlm.nih.gov/gene/) were utilized to identify targets related to DOR (44). The keyword was "diminished ovarian reserve." To enhance the credibility of the results, DOR-related targets with a gene-disease score ≥0.1 were set in DisGeNET, and the threshold of relevance score was set at 10 in GeneCards.

### Protein-Protein Interaction (PPI) Data 

The protein-protein interaction data were integrated and obtained from the Search Tool for the Retrieval of Interacting Genes (STRING) platform (https://string-db.org/) (45). The species was limited to "Homo sapiens," and the interaction confidence score was set at 0.7, defined as high confidence on the STRING platform.

### GO and KEGG Pathway Enrichment

To clarify the role of potential therapeutic targets in gene function and signaling pathways, the ClusterProfiler package of R 4.0.2 was used to perform GO and KEGG pathway enrichment analysis of the common genes for melatonin and DOR (46). The pathway class of every KEGG pathway was obtained from the KEGG PATHWAY database (https://www.kegg.jp/kegg/pathway.html) for further analysis.

### Network Construction

Five networks were constructed: (1) the melatonin-putative target network was built by connecting melatonin and its targets; (2) the PPI network of DOR targets; (3) another PPI network was constructed using the intersected melatonin and DOR genes; (4) the melatonin-targets-pathways network was established by linking melatonin, its targets, and key pathways with literature support for DOR treatment. For further analysis, the network was divided into functional modules using the Community Cluster algorithm (Glay) of clustermaker2 (47) and (5) the sub-networks of the potential therapeutic targets that were enriched in different key pathway classes were constructed. All of the above networks were established using Cytoscape 3.8.0.

### Molecular Docking Simulation

#### Target Protein Preparation

The crystal structures of the protein receptors were obtained from the RCSB Protein Data Bank (http://www.rcsb.org/). The downloaded protein structures were pretreated with PyMol 2.4.0 to remove the original ligand, solvent molecules, redundant protein chains and add polar hydrogen. Then, AutoDock Tools 1.5.6 was used to compute the Gasteiger and determine the docking box's center and size (48).

#### Ligand Preparation

The 3D structure of melatonin was treated by polarity hydrogenation and energy minimization using the MMFF94s force field.

#### Molecular Docking

AutoDock Vina was then used to evaluate melatonin binding and the hub targets by molecular docking (49). Prior to molecular docking, all protein and melatonin structures were converted to PDBQT format using AutoDock Tools 1.5.6. Melatonin was then docked onto the proteins using AutoDock Vina. Finally, the binding affinity calculated by AutoDock Vina was tallied, and the docking result was visualized using PyMol 2.4.0 software (Open-source version).

## Results

### Melatonin−Putative Target Network

**Figure 2:**
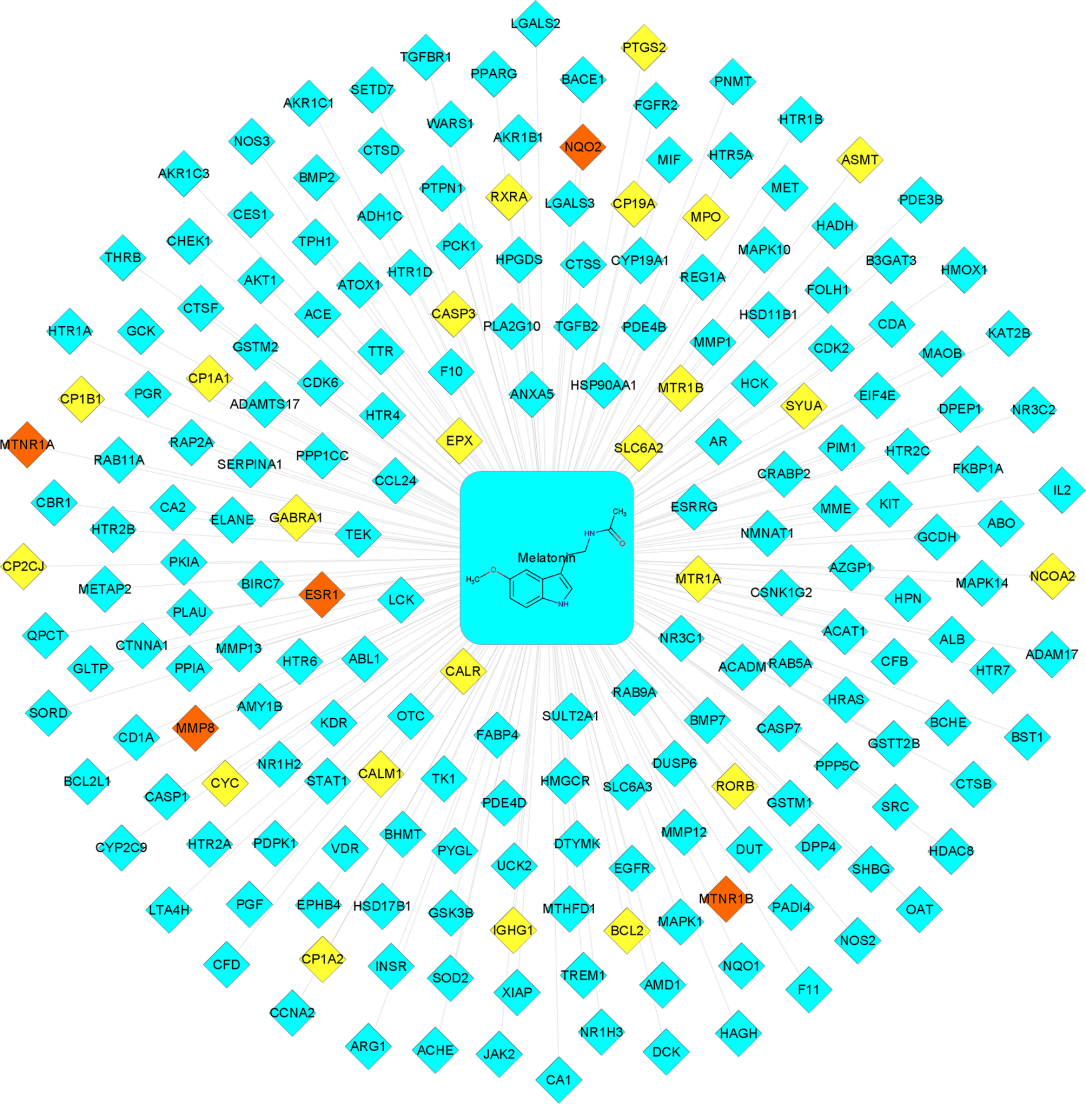
Melatonin−putative target network. The blue-colored nodes represent the potential targets. The yellow-colored nodes represent the known targets. The red-colored nodes represent the intersection of the potential and known targets.

A total of 206 melatonin targets were obtained after removing duplications from the PharmMapper, SuperTarget, DrugBank, SwissTargetPrediction, and TCMSP databases (**Supplementary 1**). Then, the melatonin-target network was constructed using Cytoscape 3.8.0 **(** **Figure 2** **)**. As shown in **Figure 2**, there were 28 known targets, accounting for 13.6% of the total targets and 183 putative targets, accounting for 88.8% of the total targets. Furthermore, there were five intersecting targets between the potential and known targets.

### PPI Network of DOR Targets

**Figure 3:**
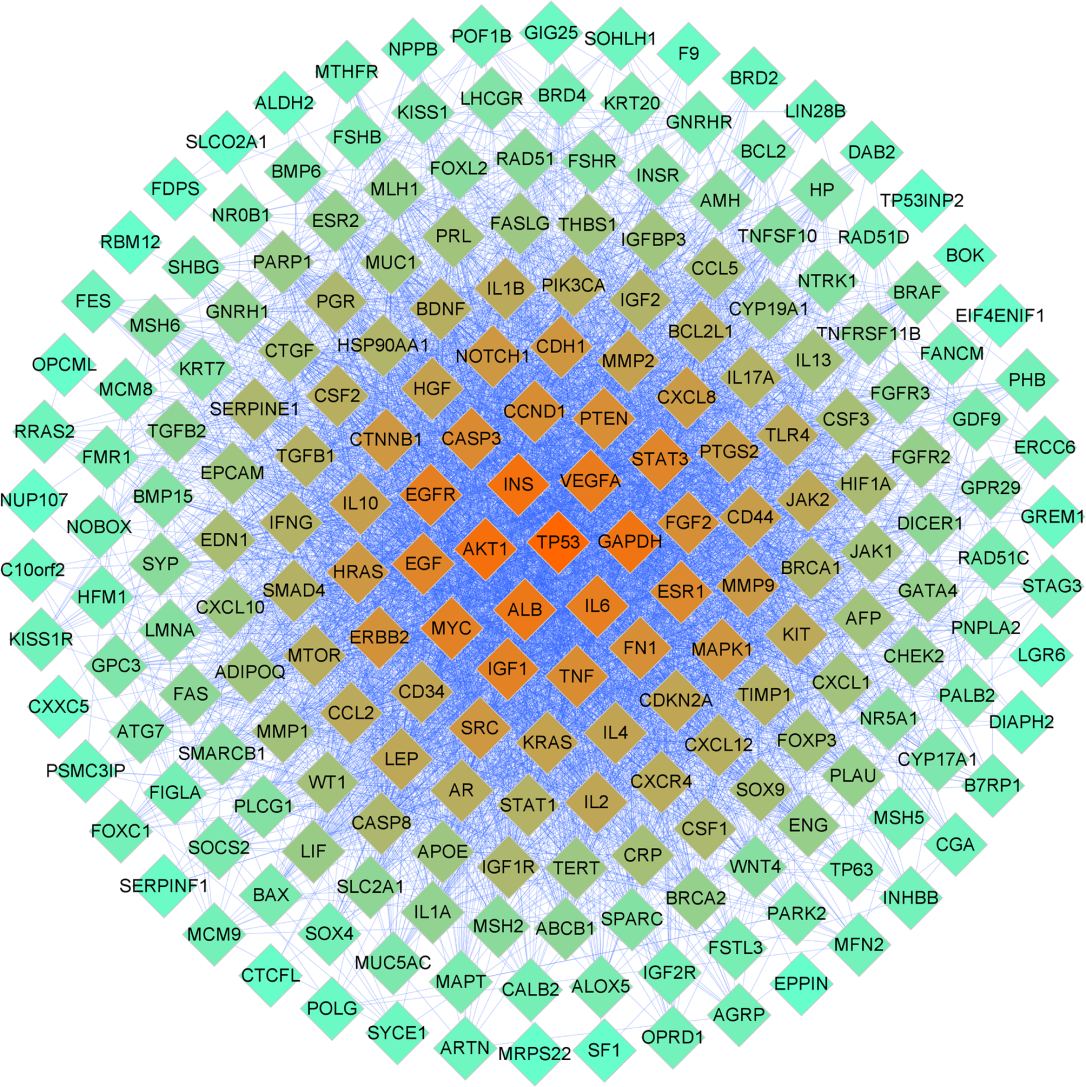
PPI network related to DOR. The color of the nodes is illustrated from red to cyan in descending order of degree values.

A total of 326 DOR-related targets were obtained from the DisGeNET, GeneCards, and NCBI Gene databases (**Supplementary 2**). A PPI network was constructed to demonstrate the interaction of DOR-related targets **(** **Figure 3** **)**. Forty-five significant DOR-related targets were obtained according to the mean values for degree centrality (DC), betweenness centrality (BC), and closeness centrality (CC), which were 47.14553991, 0.004491643, and 0.527211414, respectively (**Supplementary 3**).

### PPI Network of the Potential Therapeutic Targets

**Figure 4:**
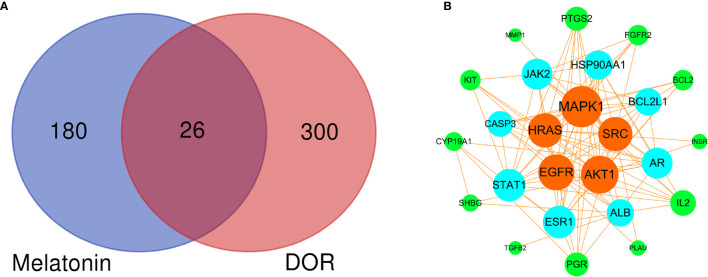
Venn diagram and PPI network of potential therapeutic targets. **(A)** Venn diagram of intersected targets of melatonin and DOR. **(B)** PPI network of potential therapeutic targets. The node sizes and colors are illustrated from large to small and orange to green in descending order of degree values.

Based on the above results, 26 common melatonin and DOR targets (potential therapeutic targets) were obtained using the Venn Diagram tool (http://bioinformatics.psb.ugent.be/webtools/Venn/) **(** **Figure 4A** and **Supplementary 4** **)**. Then, the PPI network of these 26 common targets was constructed **(** **Figure 4B** **)**. To find the hub targets in this complex biological network, the topological parameters were analyzed. As a result, there are eight hub targets in this PPI network according to DC, BC, and CC mean values, including MAPK1, AKT1, EGFR, HRAS, SRC, ESR1, AR, and ALB **(** **Supplementary 5** **)**. Meanwhile, as shown in **Table 1**, all eight hub targets were significant DOR-related targets. Therefore, these hub targets might play an essential role in OR improvement via melatonin and were used for the subsequent molecular docking study.

**Table 1:** The KEGG results.

Pathway class	Pathway	Count	Total genes	p-value
Cell growth and death	Apoptosis	6	136	2.78E-06
Endocrine and metabolic disease	AGE-RAGE signaling pathway in diabetic complications	8	100	3.79E-10
Endocrine system	Prolactin signaling pathway	7	70	1.15E-09
Endocrine system	Thyroid hormone signaling pathway	6	121	1.40E-06
Endocrine system	Growth hormone synthesis, secretion and action	5	119	2.68E-05
Endocrine system	GnRH signaling pathway	4	93	1.73E-04
Endocrine system	Ovarian steroidogenesis	3	51	4.95E-04
Endocrine system	Estrogen signaling pathway	9	138	1.55E-10
Endocrine system	Progesterone-mediated oocyte maturation	4	100	2.29E-04
Endocrine system	GnRH secretion	3	64	9.64E-04
Immune system	IL-17 signaling pathway	5	94	8.49E-06
Signal transduction	JAK-STAT signaling pathway	8	162	1.79E-08
Signal transduction	VEGF signaling pathway	5	59	8.32E-07
Signal transduction	NF-kappa B signaling pathway	4	104	2.67E-04
Signal transduction	PI3K-Akt signaling pathway	12	354	1.30E-10
Signal transduction	MAPK signaling pathway	9	294	1.22E-07
Signal transduction	FoxO signaling pathway	6	131	2.23E-06

### GO and KEGG Enrichment Analysis

#### GO Enrichment Analysis

**Figure 5:**
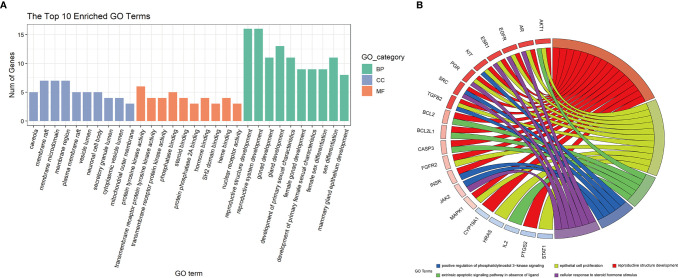
GO enrichment analysis and the top 5 enriched biological processes. **(A)** GO enrichment analysis. The top 10 significantly enriched terms of each part. BP, biological process; CC, cell component; MF, molecular function. **(B)** The top 5 enriched biological processes.

The 26 potential therapeutic targets were analyzed using the ClusterProfiler package of R 4.0.2. The top 10 terms of each part of the GO enrichment results were selected based on the counts of hit genes and the *p-*value. The results were visualized using the R package's ggplot2 and are shown in **Figure 5A**. After data screening, the top five enriched GO terms of biological processes (BP) are shown in **Figure 5B**. We could clearly identify that the top five enriched BPs of melatonin against DOR effects were mechanistically linked to reproductive structure development, epithelial cell proliferation, the extrinsic apoptotic signaling pathway, phosphatidylinositol 3-kinase signaling, and response to steroid hormones. Seven of the eight hub genes were also enriched in the top five enriched BPs, including MAPK1, AKT1, EGFR, SRC, HRAS, ESR1, and AR.

#### KEGG Enrichment Analysis of the Potential Therapeutic Targets

**Figure 6:**
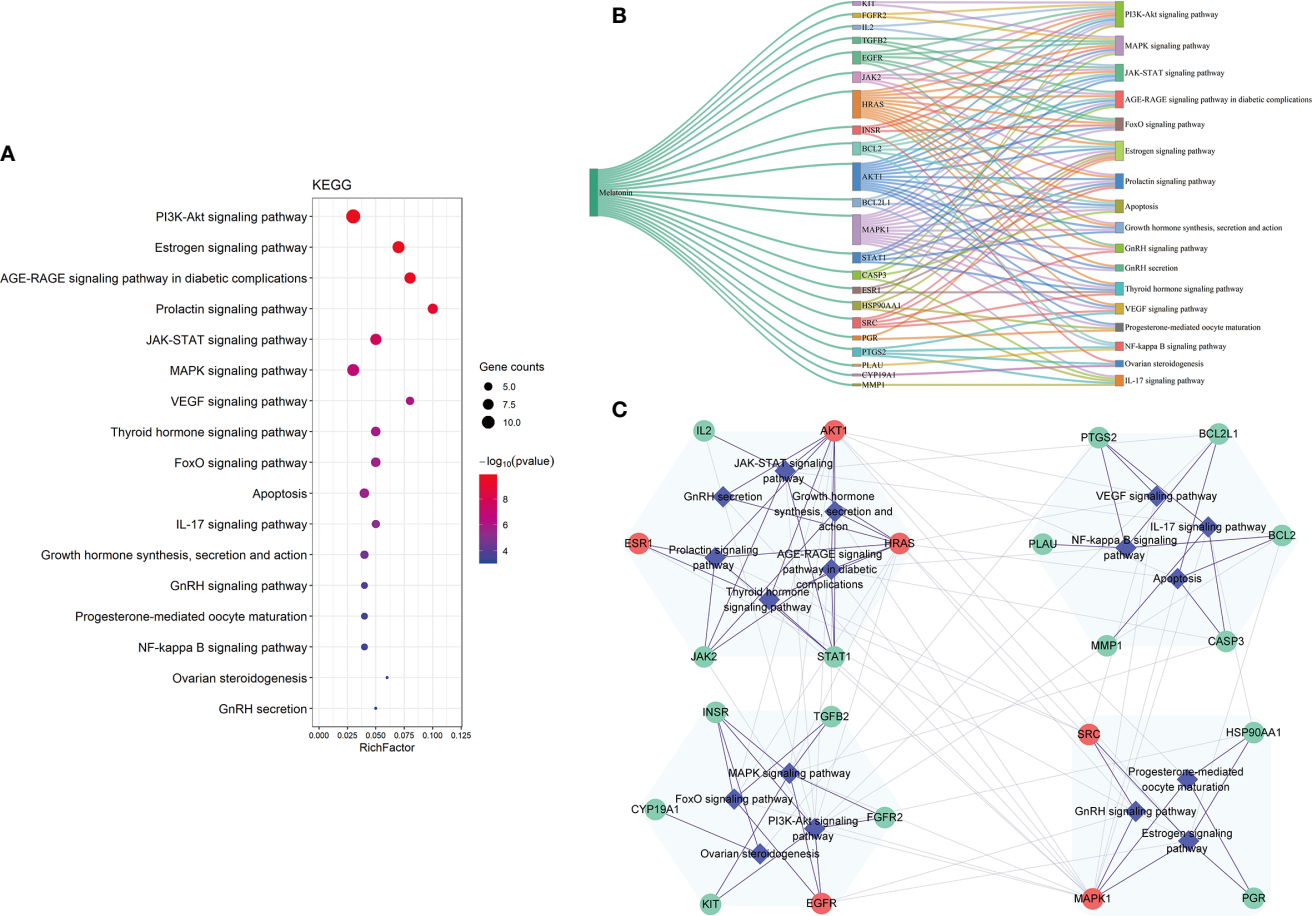
The KEGG pathway analysis of the 26 potential therapeutic targets. **(A)** The 17 significant pathways. The bubbles’ sizes are indicated from large to small in descending order of the count of the potential targets enriched in the pathways. The bubbles’ colors are indicated from red to blue in descending order of -lg (*p-*value). **(B)** Melatonin-targets-pathways network. The width of the line is proportional to the number of connected points. **(C)** Module analysis of the target-pathway network. The diamond nodes represent the pathways, and the circular nodes represent the targets. The red nodes represent the hub genes obtained from the PPI network of potential therapeutic targets.

**Figure 7:**
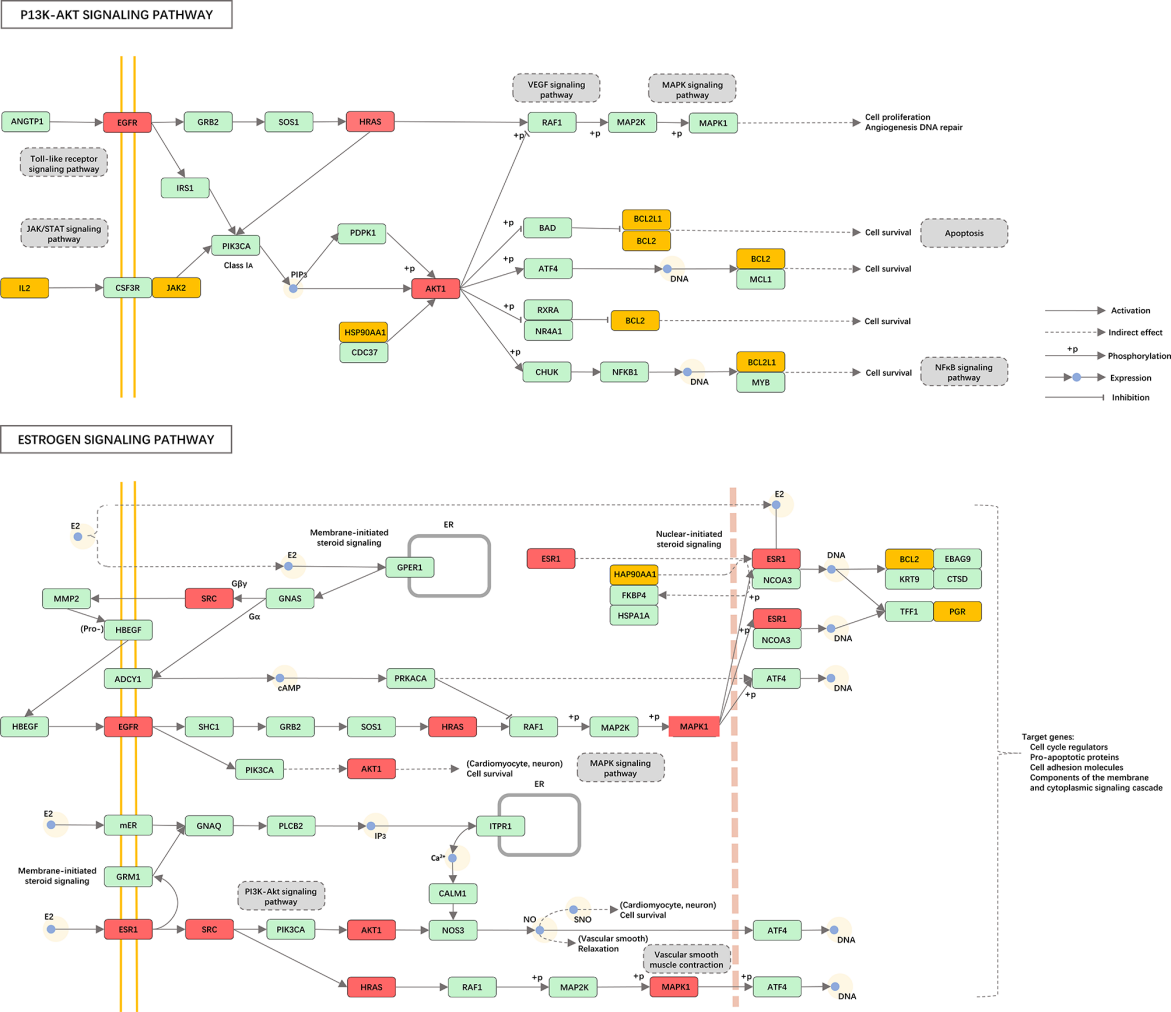
Distribution of the potential therapeutic targets on significantly enriched pathways. The red nodes represent key genes, the yellow nodes represent overlapping targets of Melatonin and DOR targets, and the green nodes represent the other targets in estrogen signaling pathway and PI3K-AKT signaling pathway.

**Figure 8:**
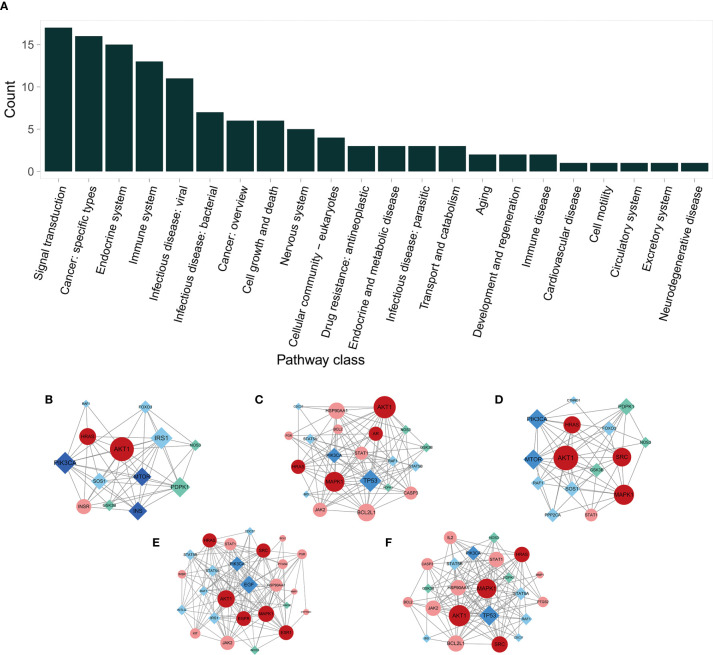
The KEGG pathway class analysis and sub-networks in different pathway classes. **(A)** The pathway class distribution. **(B–F)** Melatonin’s target sub-networks in different pathway classes. **(B)** Aging; **(C)** Cell growth and death; **(D)** Development and regeneration; **(E)** Endocrine system; and **(F)** Immune system. The circular nodes indicate the primary proteins, and the diamond nodes indicate secondary proteins. The pink nodes indicate the common targets of melatonin and DOR; the red nodes indicate the PPI network’s hub genes of potential therapeutic targets; the cyan nodes indicate the melatonin targets, and the deep blue nodes indicate the DOR targets.

We carried out KEGG pathway enrichment analysis of the 26 therapeutic targets using the ClusterProfiler package of R 4.0.2 and obtained 123 pathways with a *p-*value <0.05. After data screening, 17 significant pathways were identified **(** **Figure 6A** and **Table 1** **).** Then, the genes enriched in each pathway were sorted, and a melatonin-target-pathway network was constructed **(** **Figure 6B** **)**. In the network, the PI3K-Akt signaling pathway (hsa04151) and estrogen signaling pathway (hsa04915) were significantly enriched **(** **Figure 7** **)**. To fully understand the mechanism of melatonin in treating DOR, the target-pathway network was decomposed into functional modules using the community cluster (Glay) algorithm of clustermaker2. As illustrated in **Figure 6C**, the target pathway network was divided into four modules. Module 1 contained six pathways, including the AGE-RAGE signaling pathway in diabetic complications (hsa04933), JAK-STAT signaling pathway (hsa04630), thyroid hormone signaling pathway (hsa04919), growth hormone synthesis, secretion, and action (hsa04935), progesterone-mediated oocyte maturation (hsa04914), and GnRH secretion (hsa04929). Module 2 consisted of four pathways, including the VEGF signaling pathway (hsa04370), apoptosis (hsa04210), IL-17 signaling pathway (hsa04657), and NF-κB signaling pathway (hsa04064). Module 3 comprised four pathways, including the PI3K-Akt signaling pathway (hsa04151), MAPK signaling pathway (hsa04010), FoxO signaling pathway(hsa04068), and ovarian steroidogenesis (hsa04913). Module 4 comprised three pathways, including the estrogen signaling pathway (hsa04915), GnRH signaling pathway (hsa04912), and progesterone-mediated oocyte maturation (hsa04914). In addition, the pathway class for each of the 123 pathways in the KEGG database was obtained, and the number of KEGG pathways classified in different biological systems is shown in **Figure 8A**. Meanwhile, according to the pathway class, five sub-networks were constructed to explain melatonin's multi-mechanism on DOR integrally.

### Molecular Docking

**Figure 9:**
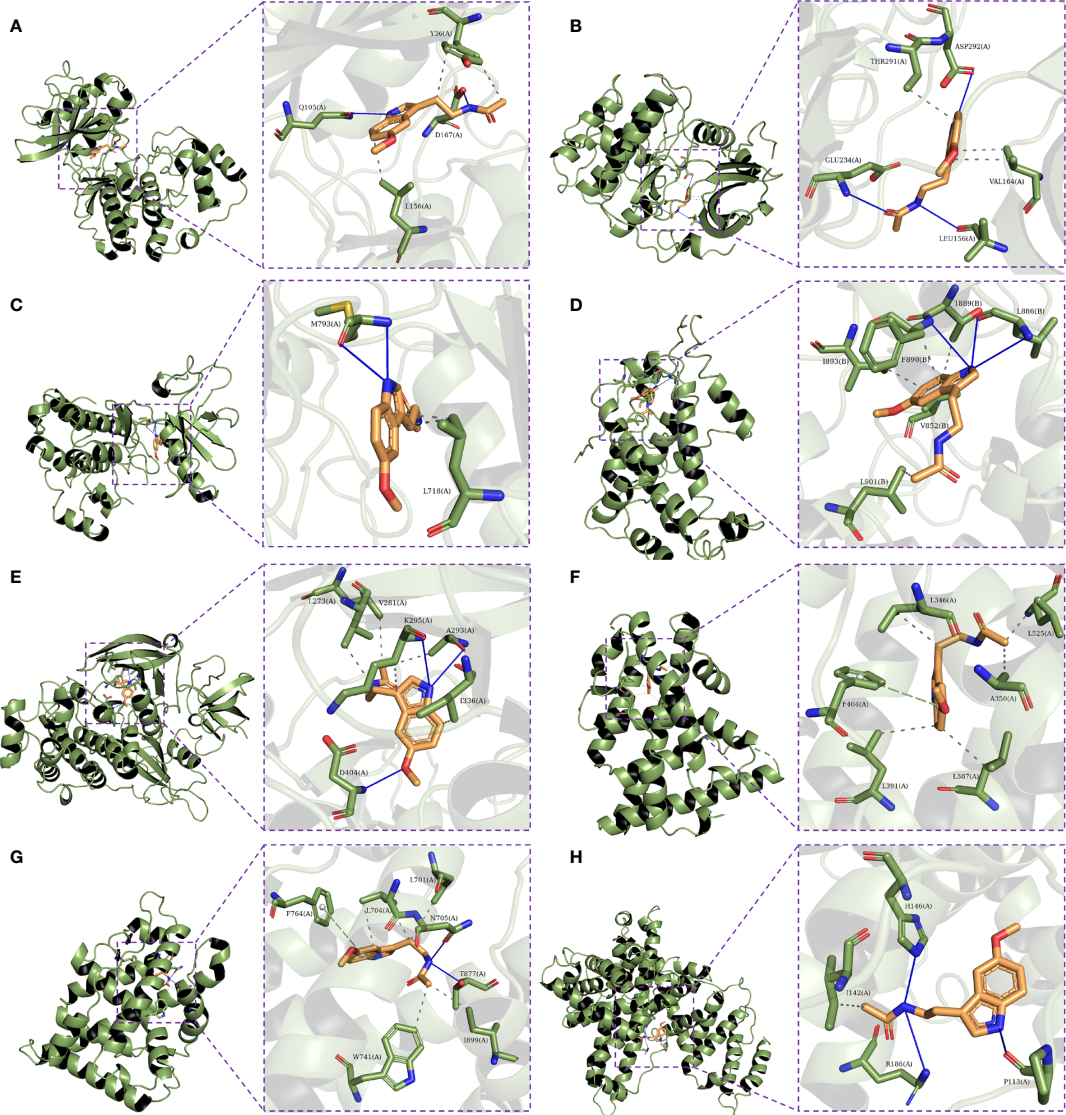
Molecular docking of the eight hub targets with Melatonin. **(A)** The binding poses of MAPK1 complexed with melatonin. **(B)** The binding poses of AKT1 complexed with melatonin. **(C)** The binding poses of EGFR complexed with melatonin. **(D)** The binding poses of HRAS complexed with melatonin. **(E)** The binding poses of SRC complexed with melatonin. **(F)** The binding poses of ESR1 complexed with melatonin. **(G)** The binding poses of AR complexed with melatonin. **(H)** The binding poses of ALB complexed with melatonin.

Eight hub genes were selected for molecular docking analysis with melatonin. The active site parameters of each target were calculated and are listed in **Table 2**. The lower docking affinity reflects the stronger binding ability between melatonin and its targets, and the binding pose with the strongest affinity was selected to analyze the interaction between melatonin and its targets. As shown in **Table 2**, except for ALB, the affinity of the remaining targets and melatonin was lower than -5 kcal/mol, indicating a strong binding affinity. Therefore, melatonin may improve OR by regulating the activity of these proteins. **Figure 9** shows the binding mode of melatonin with the hub targets. Taking **Figure 9A** as an example, melatonin completely entered the active site of AKT1 and formed hydrophobic interactions with residues T291(A) and V164(A). Moreover, the formation of three hydrogen bonds between melatonin and the active site residues of AKT1 involved residues E234 (A), L156 (A), and D292 (A).

**Table 2:** Docking parameters and results.

Targets	PDB ID	Box_center (x, y, z)/Å	Box_size (x×y×)/	Affinity/(kcal/mol)
AKT1	3MV5	5.1, 3.0, 17.9	16.4×15.4×14.7	-7.6
ALB	3JQZ	45.4, 8.9, -36.6	19.2×18.0×11.8	-4.9
AR	2PIU	27.5, 2.8, 5.5	16.4×19.3×13.0	-7.2
EGFR	2ITY	-48.9, -0.9, -22.9	21.4×16.1×22.5	-6.5
ESR1	1ERE	9.1, 46.2, 131.2	15.2×18.7×15.4	-7.3
HRAS	6D59	35.2, 30.2, 23.1	18.9×17.5×19.4	-7.1
MAPK1	5NHV	-15.6, 13.5, 42.4	19.9×16.0×15.6	-6.9
SRC	4K11	19.6, 23.1, 57.1	17.5×17.4×15.9	-7.2

## Discussion

Ovarian reserve plays a crucial role in reproductive potential and endocrine stability. Driven by societal trends, many young women choose to postpone marriage and childbirth. However, their OR sharply declines after the age of 35 years (7, 50). Besides leading to reproductive dysfunction, DOR has been associated with increased risk factors for cardiovascular disease and depression (51, 52). As mentioned previously, clinical findings have confirmed that melatonin effectively improves OR, but the therapeutic mechanism of action is still not fully understood. Therefore, in the present study, for the first time, systematic and comprehensive network pharmacology was utilized to reveal the mechanism of action of melatonin against DOR and to provide relevant information for further preclinical or clinical research. According to our network pharmacology results, AKT1, EGFR, MAPK1, HRAS, SRC, ESR1, AR, and ALB play vital roles in improving OR via melatonin. Interestingly, the molecular docking of the hub genes and melatonin exhibited high affinities, implying that the eight hub genes may be highly correlated in the treatment of DOR with melatonin.

### Melatonin’s Eight Hub Targets in DOR

AKT1, which belongs to the AKT subfamily of serine/threonine kinases, is a multifunctional protein that regulates cell growth, survival, and proliferation (53). Emerging evidence has shown that melatonin can inhibit early follicle atresia and slow down the exhaustion of the ovarian follicle reserve by regulating the PI3K/AKT pathway in mice (54). Similarly, Leung et al. found that melatonin acts as a modulator of ovarian function and stimulates theca cell steroidogenesis by activating the PI3K/AKT pathway in bovine small follicles (55). Additionally, melatonin can ameliorate decreased embryo development caused by the AKT1 inhibitor SH6 during the *in vitro* maturation step by enhancing oocyte maturation, cumulus cell expansion, and protection from DNA fragmentation (56).

EGFR also plays an essential role in ovarian function (57–59). LH-induced EGFR activation is an essential component for the communication between the outer mural granulosa and theca cells and the inner cumulus cells and oocytes, leading to cumulus cell expansion and oocyte maturation (60). Interestingly, Tian et al. found that melatonin can upregulate the expression levels of EGFR and effectively improve the efficiency of oocyte maturation *in vitro* (61). Tian et al. further showed that melatonin enhances the expression of EGFR in cumulus cells and improves cumulus-oocyte complex maturation, mainly via melatonin receptor 1 (62). Moreover, several studies have shown that the activation of EGFR promotes several signaling pathways, including MAPK, PI3K/AKT, and JAK/STAT pathways, all of which play a crucial role in follicle recruitment, development, and maturation (63–66). These results suggest that melatonin enhances ovarian reserve variously by upregulating EGFR levels.

MAPK1, also known as extracellular signal-regulated kinase 2 (ERK2), is a downstream effector of the EGFR pathway. Activated ERK regulates the expression of LHβ and FSHβ, which are gonadotropin synthesis genes, and induces follicle growth and ovulation (67, 68). In addition, the activation of EGFR-ERK1/2 dependent gene transcription leads to the cascade of prostaglandin E2 and p38MAPK induction, which in turn stimulates the production of EGFR ligands (AREG, EREG, and BTC) in granulosa and cumulus cells, finally activating the entire EGF network (58). For the first time (2001), Leung et al. found that melatonin via the MAPK pathway regulates progesterone production, LH receptor, GnRH, and GnRH receptor gene expression in human granulosa-luteal cells, which play a direct role in regulating ovarian function (69). Furthermore, melatonin has been shown to enhance follicle growth and proliferation in cadmium-induced injury in rat ovaries via the ERK1/2 and mTOR pathways (70).

As the primary female sex hormones, estrogens are responsible for maturing and maintaining the female reproductive system and are also involved in gonadotropin secretion and ovarian follicle maturation. Estrogens exert their functions by binding to ERα and ERβ, encoded by ESR1 and ESR2, respectively. SRC and HRAS, the downstream proteins of ESR1 in the estrogen signaling pathway, participate in various cellular processes, including proliferation, differentiation, and adhesion (71). Many studies support the beneficial effects of androgens in follicular development, which may be related to AR upregulating FSH receptor expression, stimulating FSH activity in GCs, and promoting follicles from the anterior sinus phase to the anal phase (72–75). In addition, although the expression pattern and role of ALB in the ovaries have not been fully clarified, as a major serum protein, ALB plays a vital role in steroid hormone (SHs) carriers and acts as a regulator of SHs' access to their receptors (76, 77). At present, no animal or clinical studies have directly confirmed that melatonin can improve OR through the above five targets (ESR1, HRAS, SRC, AR, and ALB). However, based on the molecular docking results in the current study (as well as AKT1, MAPK1, and EGFR, the above five targets have a good binding ability with melatonin) and combining their physiological roles in the ovaries, we speculate that melatonin could play a beneficial role in ovaries via these targets. These results provide a preliminary basis and reference for future in-depth research on the mechanism of melatonin in animal models.

### Important Pathways and Functional Modules of Melatonin’s Putative Targets

The pathophysiological mechanism of DOR is especially complicated, and various biological processes and pathways are involved in the DOR process. The 26 therapeutic targets screened in this study mainly participate in reproductive structure development, epithelial cell proliferation, the extrinsic apoptotic signaling pathway, PI3K signaling, and response to steroid hormones. Furthermore, the KEGG pathway analysis indicated that the PI3K-Akt signaling pathway (hsa04151) and the estrogen signaling pathway (hsa04915) were the two most enriched signaling pathways (**Figure 6A**). Accumulating evidence suggests that the PI3K-Akt signaling pathway plays a key role in folliculogenesis processes, including follicle recruitment, development, and maturation (63–65). The estrogen signaling pathway is vital for the maturation and maintenance of the female reproductive system (78).

To further understand melatonin mechanisms in improving OR, the target pathway network was divided into four densely linked functional modules, as shown in **Figure 6C**. The 1st module consists of pathways in the endocrine system and related signaling pathways. The 2nd module includes pathways in cell growth and death, the immune system, and related signaling pathways, and the 3rd module is related to signal transduction. The 4th module includes pathways in the endocrine system related to the regulation of ovarian function. According to the theory of network biology, the topology of a biological network is bridged to its function (79). These modules reflected melatonin's effects on endocrine and immune regulation, anti-apoptosis, and ovarian function improvement. In addition, exogenous growth hormone administration has been shown to improve oocyte and embryo quality in IVF treatment of women with poor OR (80, 81). The functional modules analysis showed that melatonin is closely related to the synthesis, secretion, and action of GH, which also supports the function of melatonin in improving OR.

### Biological Processes and Organ Systems Regulated by Melatonin's Putative Targets

Importantly, in this study, to explain the multi-mechanism of melatonin on DOR, five sub-networks were constructed. The aging sub-network (**Figure 8B**) showed that melatonin targets AKT1, mTOR, and PIK3A, among others. The AKT/TOR pathway is a recognized central signaling pathway regulating lifespan, highlighting the anti-aging effect of melatonin (82, 83). Consistently, previous studies have shown that melatonin can prolong the lifespan and delay ovarian aging in mice  (84, 85).

Apoptosis is a critical biological process that plays a vital role in germ cell depletion in mammalian ovaries (86). Follicular atresia caused by GC apoptosis is the primary process responsible for follicle loss (87–89). Bcl2-like-proteins are anti-apoptotic factors that may inhibit apoptosis. In the subnetwork of cell growth and death (**Figure 8C**), melatonin acts on BCL2, BCL2L1, BID, and CASP3, suggesting that melatonin exerts anti-apoptotic effects.

The sub-network of development and regeneration (**Figure 8D**) suggests the effect of melatonin in follicle development regulation. This network includes AKT, MAPK1, and HARS, which are involved in follicle growth and survival (90).

In addition to maintaining homeostasis, the immune system is associated with modulation at every level of the hypothalamic-pituitary-ovarian axis, as well as the regulation of proliferation and differentiation of ovarian germline stem cells (91). AKT1 and its interactions with MAPK1, JAK2, STAT1, etc., are involved in regulating melatonin in both the endocrine and immune systems (**Figures 8E, F**). Although they are not immune genes, they play an essential role in the division, differentiation, development, and function of various types of immune genes and immunomodulatory cytokines, including T-cells, IFN, Th17, and dendritic cells (92–94).

## Conclusions

In summary, melatonin may improve OR by intervening in a series of targets (such as AKT1, EGFR, MAPK1, HRAS, SRC, ESR1, AR, and ALB), biological processes (reproductive structure development, epithelial cell proliferation, extrinsic apoptotic signaling pathway, PI3K signaling, and response to steroid hormone), and signaling pathways (such as PI3K-Akt and estrogen signaling pathways). Melatonin could exhibit anti-aging, anti-apoptosis, endocrine, and immune system regulation.

## Data Availability Statement

The raw data supporting the conclusions of this article will be made available by the authors, without undue reservation.

## Author Contributions

QZ conceptualized the manuscript. LY, HX, YC, YX, CM, and YZ collected the literature, wrote the manuscript, and made the figures. QZ edited and made significant revisions to the manuscript. All authors contributed to the article and approved the submitted version.

## Funding

This work was financially supported through grants from the National Natural Science Foundation of China (81904241), the Program of Zhejiang Provincial TCM Sci-tech Plan (2020ZA078), the Medical and Health Science and Technology Plan of Zhejiang Province (2021KY920), and the Zhejiang Zhangqin Famous Traditional Chinese Medicine Expert Inheritance Studio Project (GZS2012014).

## Conflict of Interest

The authors declare that the research was conducted in the absence of any commercial or financial relationships that could be construed as a potential conflict of interest.

## Abbreviations

AKT1, RAC-alpha serine/threonine-protein kinase; ALB, Albumin; AR, Androgen receptor; ART, Assisted reproductive technology; BC, Betweenness centrality; BP, Biological process; CC, Closeness centrality; DC,Degree centrality; DOR, Diminished ovarian reserve; EGFR, Epidermal growth factor receptor; ESR1, Estrogen receptor; GO, Gene Ontology; HRAS, GTPase HRas; KEGG, Kyoto Encyclopedia of Genes and Genomes; MAPK1, Mitogen-activated protein kinase 1; OR, Ovarian reserve; PPI, Protein–protein interaction; SMILES, Simplified molecular input entry specification; SRC, Proto-oncogene tyrosine-protein kinase Src; STRING, The Search Tool for the Retrieval of Interacting Genes; TCMSP, Traditional Chinese Medicine Systems Pharmacology Database and Analysis Platform; Uniprot, Universal Protein.
